# Clinical characteristics and risk factors for 30-day mortality in esophageal cancer patients with upper gastrointestinal bleeding: a multicenter study

**DOI:** 10.3389/fonc.2023.1184710

**Published:** 2023-05-04

**Authors:** Sz-Wei Lu, Chu-Pin Pai, Ting-Hao Yang, Jian-Xun Lu, Chien-Han Hsiao, Chieh-Ching Yen

**Affiliations:** ^1^ Department of Emergency Medicine, Keelung Chang Gung Memorial Hospital, Keelung, Taiwan; ^2^ Department of Emergency Medicine, Tri-Service General Hospital SongShan Branch, National Defense Medical Center, Taipei, Taiwan; ^3^ Division of Thoracic Surgery, Department of Surgery, Luodong Poh-Ai Hospital, Ilan, Taiwan; ^4^ Department of Emergency Medicine, Chang Gung Memorial Hospital, Linkou Branch, Taoyuan, Taiwan; ^5^ Department of Linguistics, Indiana University, Bloomington, IN, United States; ^6^ Department of Emergency Medicine, New Taipei Municipal Tucheng Hospital, New Taipei City, Taiwan

**Keywords:** esophageal cancer, etiology, gastrointestinal bleeding, Outcome, Mortality

## Abstract

**Background:**

Esophageal cancer is a highly malignant neoplasm with poor prognosis. Of its patients, upper gastrointestinal bleeding (UGIB) is one of the most challenging and threatening conditions in the emergency department (ED). However, no previous studies have analyzed the etiologies and clinical outcomes in this specific population. This study aimed to identify the clinical characteristics and risk factors for 30-day mortality in esophageal cancer patients with UGIB.

**Methods:**

This retrospective cohort study enrolled 249 adult patients with esophageal cancer presenting with UGIB in the ED. Patients was divided into the survivor and non-survivor groups, and their demographic information, medical history, comorbidities, laboratory parameters, and clinical findings were recorded. The factors associated with 30-day mortality were identified using Cox’s proportional hazard model.

**Results:**

Among the 249 patients in this study, 30-day mortality occurred in 47 patients (18.9%). The most common causes of UGIB were tumor ulcer (53.8%), followed by gastric/duodenal ulcer (14.5%), and arterial-esophageal fistula (AEF) (12.0%). Multivariate analyses indicated that underweight (HR = 2.02, *p* = 0.044), history of chronic kidney disease (HR = 6.39, *p* < 0.001), active bleeding (HR = 2.24, *p* = 0.039), AEF (HR = 2.23, *p* = 0.046), and metastatic lymph nodes (HR = 2.99, *p* = 0.021) were independent risk factors for 30-day mortality.

**Conclusions:**

The most common cause of UGIB in esophageal cancer patients was tumor ulcer. AEF, accounting for 12% of UGIB in our study, is not an uncommon cause. Underweight, underlying chronic kidney disease, active bleeding, AEF, and tumor N stage > 0 were independent risk factors for 30-day mortality.

## 1 Introduction

Esophageal cancer is the ninth most common cancer and the sixth most frequent leading cause of cancer deaths globally (1–3). The reported 5-year survival rate for patients with esophageal cancer ranges from 15% to 25%, and its unfavorable outcome are attributable to the fact that esophageal cancer is usually diagnosed at an advanced stage with a high propensity for metastases (1–3). The histological types of squamous cell carcinoma (SCC) and adenocarcinoma (ADC) account for 90% of esophageal cancer worldwide. The prevalent histological type and the incidence rate of esophageal cancer vary in different geographic areas. In the “Asian Esophageal Cancer Belt”, which ranges from Northeast China to Middle East, SCC esophageal cancer has a high incidence rate of more than 100 cases per 100000 people (4). Although Taiwan is not included in this geographic region, SCC is also the main histological type (5).

Upper gastrointestinal bleeding (UGIB) accounts for 5.7% of the total emergency department (ED) visits of the esophageal cancer patients (6). UGIB usually presents as melena or hematemesis and is a life-threatening condition requiring urgent treatments. Traditionally, UGIB was managed by endoscopic treatment, arteriography with embolization, or surgical intervention, and its prognosis was evaluated with clinical assessment and endoscopic findings (7). When encountering esophageal cancer patients with UGIB, cancer-related complications like tumor bleeding, fistula formation, or post-operative leakage should be considered in addition to the common causes of UGIB, e.g., peptic ulcer disease. The early recognition of these life-threatening diseases is essential for the prompt initiation of an appropriate treatment to achieve favorable outcomes (8, 9). However, the etiology and clinical outcome of UGIB in patients with esophageal cancer have not been reported in the literature. Therefore, we aimed to examine the clinical characteristics and risk factors for 30-day mortality in esophageal cancer patients with UGIB.

## 2 Materials and methods

### 2.1 Study design and setting

This was a retrospective multiple-center observational study conducted with the data from the emergency departments (ED) of five hospitals sharing the same electronic medical records (EMRs) system in Taiwan. The study sites had over 9,000 beds and approximately 500,000 ED visits annually. All adult patients who met the inclusion criteria of the study from 1 January, 2016 to 31 May, 2022 were retrospectively enrolled for analysis. The mortality in the study was assessed at 30 days post-ED presentation. The current study was approved by the Chang Gung Medical Foundation Institutional Review Board (IRB no. 202201321B0) and was performed in accordance with the Declaration of Helsinki.

### 2.2 Patient selection and data collection

Through a computerized search of the EMRs during the study period, all adult patients with esophageal cancer who presented with UGIB and were treated at the EDs were identified with the International Classification of Diseases (ICD)-10 codes C15 (Malignant neoplasm of esophagus) and K920-K922 (Hematemesis, Melena, Gastrointestinal hemorrhage, unspecified). The source of bleeding was localized by endoscopy or image reports. UGIB was defined as bleeding originated from proximal to the ligament of Treitz (10). Patients were excluded if they had incomplete medical records, duplicated ED visit record, or bleeding from sites inconsistent with the UGIB definition, e.g., hemorrhoids, small intestine, or colon. The records of the patients selected with EMRs were further reviewed by two physicians for the verification of their inclusion eligibility (S-WL and C-PP).

For each patient, the baseline characteristics of sex, age, body mass index (BMI), lifestyle factors (cigarette, betel nut and alcohol use), initial ED vital signs and presenting symptoms, drug history, comorbidities (i.e., hypertension, diabetes mellitus, coronary artery disease, chronic kidney disease, chronic obstructive pulmonary disease, other malignancy, prior stroke, gastroesophageal reflux disease, and liver cirrhosis), and the grade on the performance status scale by Eastern Cooperative Oncology Group (ECOG-PS) were retrieved (11). The initial presenting symptoms included hematemesis, melena, and hematochezia. However, patients with hematochezia were excluded because bleeding from lower gastrointestinal site was noted in all of them on the endoscopy or image reports in our study. Active bleeding was defined as patients with continued bleeding at the ED. The collected laboratory data at the initial presentation included white blood cell count (WBC), hemoglobin (Hb), platelet count (PLT), prothrombin time (PT), blood urea nitrogen (Bun), creatinine, and alanine aminotransferase (ALT). The clinicopathological parameters of the primary cancer were obtained based on the most recent available data at the time of bleeding occurrence. These included information pertaining the cancer site, tumor-node-metastasis (TNM), stage (based on the TNM staging system by the American Joint Committee on Cancer, 8^th^ edition) (12), cancer treatment modality, and local recurrence.

Emergent endoscopy or computed tomography (CT) angiography was performed to determine the cause of bleeding and localized the bleeding site. The etiology of esophageal cancer patients with UGIB consisted of tumor ulcer, arterial-esophageal fistula (AEF), esophageal or gastric varices, gastric or duodenal ulcer, gastritis or duodenitis, esophageal ulcer, and post-operative complications, which included anastomotic leakage or ruptured. Endoscopy was the primary imaging modality for most treating physicians when managing esophageal cancer patients with UGIB in our institutions. However, in some cases, emergent endoscopy was unavailable for unstable patients during their initial ED visit. As an alternative, these patients were managed with emergent CT angiography. Some patients underwent CT angiography during their treatment in the ED, while others received the procedure during their hospital stay in the wards. CT angiography was typically performed under the following circumstances: patients presenting with active, life-threatening bleeding; patients with unstable hemodynamic status when endoscopic examination was unavailable; or cases where endoscopic treatment had failed. The treatment options included supportive care, endoscopic therapy, arterial embolization, stent implantation, and surgical intervention. Supportive care was defined as medication with intravenous proton pump inhibitors (PPI), terlipressin or tranexamic acid. Endoscopic therapy consisted of the use of argon coagulation, hemoclip, epinephrine injection, and band ligation. Patients who required inotropic support, intubation and intensive care units (ICU) admission were also recorded. The primary outcome was the presence/absence of mortality within 30 days of the initial bleeding presentation at the ED.

### 2.3 Statistical analysis

Patient characteristics, previous medical history, laboratory findings, and presentations of cancer and bleeding were presented as numbers and percentages for the categorical variables, while the continuous data were presented as mean ± standard deviation (SD). Comparisons between survivors and non-survivors were examined with Chi-square test or Fisher’s exact test for the categorical variables, and independent Student’s t-test or Mann-Whitney U-test for the normally distributed and the skewed continuous variables, respectively. Kaplan-Meier analysis was performed to assess the cumulative 30-day survival curve. To identify the independent risk factors for 30-day mortality, we employed a stepwise approach. Univariate analyses were first performed to identify the variables that are potentially associated with 30-day mortality. Second, all significant variables in the univariate analyses were input into the multivariate Cox proportional hazards model to further examine their statistical association with 30-day mortality. A subgroup analysis on active bleeding was also performed in our study. The statistics analyses were performed in SPSS software v26 (SPSS Inc., Chicago, IL). A two-sided *p* value of < 0.05 was considered the threshold of statistical significance.

## 3 Results

### 3.1 Patient characteristics of survivors and non-survivors

A total of 249 patients met the inclusion criteria of this study. 30-day mortality occurred in 47 (18.9%) patients. Patient characteristics, including initial vital signs and laboratory findings, are shown in **Table 1**. In total, 181 (72.6%) patients were admitted to ordinary wards, 31 (12.4%) were transferred to the ICU from the ED or wards, 27 (10.8%) were discharged from the ED, and 10 (4%) died in the ED. Male accounted for the majority of the patients in this study (n = 240, 96.4%), and the average age was 59.5 ± 10.7 (range: 38-90 years). The non-survivor group had a significantly lower average BMI (19.9 vs. 21.3, *p* = 0.034) compared to the survivor group. The distributions of age, sex and initial vital signs (i.e., blood pressure, heart rate, and respiratory rate) did not differ between the survivors and the non-survivors. Most underlying disease had similar proportion in both groups, except that a history of chronic kidney disease (CKD) (21.2% vs. 4.9%, *p* = 0.001) was significantly higher in the non-survivors. The initial laboratory parameters were evaluated, and the patients who did not survive displayed higher WBC (10^3^/μl) (14.2 vs. 9.4, *p* = 0.004), BUN (mg/dL) (34.4 vs. 24.8, *p* = 0.013) and creatinine (mg/dL) (1.35 vs. 1.05, *p* = 0.042) levels and a lower Hb (g/dL) (8.7 vs. 9.5, *p* = 0.046) level.

**Table 1:** Clinical and demographic characteristics of study population by 30-day mortality.

Variable	Total N=249	Survivor N=202	Non-survivor N=47	P value
Age (years)	59.5 ± 10.7	58.9 ± 10.1	62.0 ± 13.0	0.140
Male	240 (96.4)	195 (96.5)	45 (95.7)	0.679
BMI (kg/m^2^)	21.0 ± 3.6	21.3 ± 3.6	19.9 ± 4.1	0.034^*^
**ECOG-PS**				0.020^*^
0	6 (2.4)	5 (2.4)	1 (2.1)	
1	188 (75.5)	160 (79.2)	28 (59.5)	
2	33 (13.2)	24 (11.9)	9 (19.1)	
3	18 (7.2)	11 (5.4)	7 (14.8)	
4	4 (1.6)	2 (0.9)	2 (4.2)	
Initial Vital Signs				
SBP (mmHg)	120.1 ± 26.8	121.4 ± 26.4	114.5 ± 28.2	0.118
DBP (mmHg)	75.3 ± 15.0	76.1 ± 15.0	72.0 ± 15.1	0.099
Heart rate (beats/min)	107.3 ± 20.9	106.6 ± 21.2	110.4 ± 19.2	0.254
Respiratory rate (breaths/min)	19.1 ± 2.7	19.0 ± 2.6	19.7 ± 3.1	0.109
**Shock index (SI)**	0.9 ± 40.31	0.92 ± 0.29	1.04 ± 0.38	0.028^*^
Personal Habits				
Smoking history	210 (84.3)	173 (85.6)	37 (78.7)	0.266
Betel nut chewer	129 (51.8)	107 (52.9)	22 (46.8)	0.518
Alcohol consumption	189 (75.9)	158 (78.2)	31 (65.9)	0.890
Comorbidity				
Hypertension	78 (31.3)	61 (30.1)	17 (36.1)	0.485
Diabetes mellitus	45 (18.0)	38 (18.8)	7 (14.8)	0.675
Coronary artery disease	11 (4.4)	9 (4.4)	2 (4.2)	1.000
Congestive heart failure	4 (1.6)	4 (1.9)	0 (0)	1.000
Gastroesophageal reflux disease	151 (60.6)	122 (60.3)	29 (61.7)	1.000
Chronic kidney disease	20 (8.0)	10 (4.9)	10 (21.2)	0.001^*^
Prior cerebrovascular accident	13 (5.2)	8 (3.9)	5 (10.6)	0.075
Liver cirrhosis	58 (23.2)	50 (24.7)	8 (17.0)	0.339
Chronic obstructive pulmonary disease	6 (2.4)	5 (2.4)	1 (2.1)	1.000
Other malignancy	69 (27.7)	60 (29.7)	9 (19.1)	0.154
**Charlson comorbidity index (CCI)**	7.06 ± 2.88	6.78 ± 2.84	8.26 ± 2.77	0.001^*^
Current Medication				
Use of NSAIDs	31 (12.4)	22 (10.8)	9 (19.1)	0.141
Use of Anti-platelets Agent^*^	10 (4.0)	2 (4.2)	8 (4.0)	1.000
Use of Anti-coagulant Agent**^†^**	6 (2.4)	1 (2.1)	5 (2.8)	1.000
Use of PPI or H2-receptor antagonist	103 (41.3)	93 (46.0)	10 (21.2)	0.746
**Initial Presenting symptoms**				0.063
Hematemesis	160 (64.2)	124 (61.3)	36 (76.5)	
Melena	89 (35.7)	78 (38.6)	11 (23.4)	
**Active bleeding**	62 (24.9)	38 (18.8)	24 (51.0)	<0.001^*^
Initial Laboratory data				
WBC (10^3^/μL)	10.3 ± 9.0	9.4 ± 8.5	14.2 ± 10.1	0.004^*^
Hb (g/dl)	9.4 ± 2.6	9.5 ± 8.7	8.7 ± 2.2	0.046^*^
PLT (10^3^/μL)	232 ± 138	230 ± 140	239 ± 129	0.666
PT (sec)	12.86.2	12.86.7	13.23.5	0.668
Bun (mg/dL)	26.6 ± 18.6	24.8 ± 17.1	34.4 ± 22.8	0.013^*^
Creatinine (mg/dL)	1.10 ± 0.90	1.05 ± 0.89	1.35 ± 0.90	0.042^*^
ALT (U/L)	36.7 ± 58.8	32.6 ± 33.4	54.1 ± 114.6	0.219

Count data are expressed as number (percentage) and continuous values are expressed as mean ± SD.

ECOG-PS, Eastern Cooperative Oncology Group Performance Score; NSAIDsl Non-Steroidal Anti-Inflammatory Drug; BMI, body mass index; PPI, Proton pump inhibitor; SBP, systolic blood pressure; DBP, diastolic blood pressure; WBC, white blood cell; Hb, hemoglobin; PLT, platelet count; PT, prothrombin time; ALT, alanine aminotransferase; Bun, Blood urea nitrogen; CRP, C-Reactive Protein.

Anti-platelet agents including Aspirin (9) or Clopidogrel (2).

†Anti-coagulant agents including Warfarin(1), Apixban(2), Rivaroxaban(3).

*P value<0.05.

The characteristics of esophageal cancer are presented in **Table 2**. Squamous cell carcinoma (n = 227, 91.1%) was the most common histological type in this study. More than half of the patients (n = 223, 89.6%) had locally advanced esophageal cancer, and local recurrence was noted in 74 (29.7%) patients.

**Table 2:** Characteristics of tumor and clinical outcomes of the study papulation by 30-day mortality.

Variable	Total N=249	Survivor N=202	Nonsurvivor N=47	P value
**Tumor site (Esophagus)**				0.682
Upper third	56 (22.4)	47 (23.2)	9 (19.1)	
Middle third	102 (40.9)	80 (39.6)	22 (46.8)	
Lower third	91 (36.5)	75 (37.0)	16 (34.0)	
**Tumor length (cm)**	6.23±3.27	6.15±3.27	6.55±3.28	0.487
**Tumor pathology**				0.014^*^
Squamous cell carcinoma	227 (91.1)	189 (93.5)	38 (80.8)	
Adenocarcinoma	13 (5.2)	9 (4.4)	4 (8.5)	
Small cell carcinoma	2 (0.8)	1 (0.4)	1 (2.1)	
Melanoma	1 (0.4)	0 (0)	1 (2.1)	
Neuroendocrine	1 (0.4)	0 (0)	1 (2.1)	
Unknown	5 (2.0)	3 (1.4)	2 (4.2)	
**T stage**				0.004^*^
T1	28 (11.2)	27 (13.3)	1 (2.1)	
T2	26 (10.4)	17 (8.4)	9 (19.1)	
T3	112 (44.9)	89 (44.0)	23 (27.6)	
T4	78 (31.3)	67 (33.1)	11 (23.4)	
Unknown	5 (2.0)	2 (0.9)	3 (6.3)	
**N stage**				0.003^*^
N0	46 (18.4)	42 (20.7)	4 (8.5)	
N+	199 (79.9)	159 (78.7)	40 (85.1)	
Unknown	4 (1.6)	1 (0.4)	3 (6.3)	
**M stage**				0.033^*^
M0	172 (69.0)	145 (71.7)	27 (57.4)	
M1	74 (29.7)	56 (27.7)	18 (38.2)	
Unknown	3 (1.2)	1 (0.4)	2 (4.2)	
**Cancer stage**				0.013^*^
I	26 (10.4)	26 (12.8)	0 (0)	
II	21 (8.4)	15 (7.4)	6 (12.7)	
III	62 (24.8)	47 (23.2)	15 (31.9)	
IV	140 (56.2)	114 (56.4)	26 (55.3)	
Initial cancer treatment				
Surgical resection	45 (18.0)	39 (19.3)	6 (12.7)	0.400
Chemoradiation	191 (76.7)	154 (76.2)	37 (78.7)	0.849
Stent implantation	47 (18.9)	37 (18.3)	12 (25.5)	0.308
**Local recurrence**	74 (29.7)	53 (26.2)	21 (51.0)	0.014^*^
Bleeding cause				
Tumor ulcer	134 (53.8)	109 (53.9)	25 (53.1)	1.000
Arterial-esophageal fistula	30 (12.0)	16 (7.9)	14 (29.7)	<0.001^*^
Esophageal/Gastric varices	12 (4.8)	11 (5.4)	1 (2.1)	0.473
Gastric/Duodenal ulcer	36 (14.5)	33 (16.3)	3 (8.5)	0.616
Gastritis/Duodenitis	12 (4.8)	12 (5.9)	0 (0)	0.130
Esophageal ulcer	13 (5.2)	12 (5.9)	1 (2.1)	0.472
Post-operative complication	12 (4.8)	9 (4.4)	3 (6.3)	0.703
Emergent examination				
Endoscopy	220 (88.3)	183 (90.6)	37 (78.7)	0.022^*^
Emergent CTA	46 (18.4)	31 (15.3)	15 (31.9)	0.012^*^
Bleeding treatment				
Proton pump inhibitor	234 (93.9)	189 (93.6)	45 (95.7)	0.743
Tranexamic acid	161 (64.7)	125 (61.9)	36 (76.6)	0.064
Terlipressin	42 (16.9)	37 (18.3)	5 (10.6)	0.280
Endoscopic treatment**^†^**	31 (12.4)	26 (12.8)	5 (10.6)	1.000
Surgical repair/Stent implantation	26 (10.4)	20 (9.9)	6 (12.7)	0.597
Arterial embolization	8 (3.2)	7 (3.4)	1 (2.1)	0.711
**Blood transfusion**	147 (59.0)	109 (53.9)	38 (80.8)	0.001^*^
**Intubation**	30 (12.0)	17 (8.4)	13 (27.6)	0.001^*^
**Inotropic agents support**	7 (2.8)	2 (0.9)	5 (10.6)	0.003^*^
**ICU admission**	31 (12.4)	23 (11.3)	8 (4.7)	0.064
**Hospice care**	76 (30.5)	58 (28.7)	18 (38.3)	0.220

Count data are expressed as number (percentage) and continuous values are expressed as mean ± SD.

CTA, computed tomography angiography; ICU, intensive care units.

†Endoscopic treatment included use of Argon coagulation, Hemoclip, Epinephrine injection and Band ligation.

*P value<0.05.

### 3.2 Bleeding and various treatment modalities

**Figure 1:**
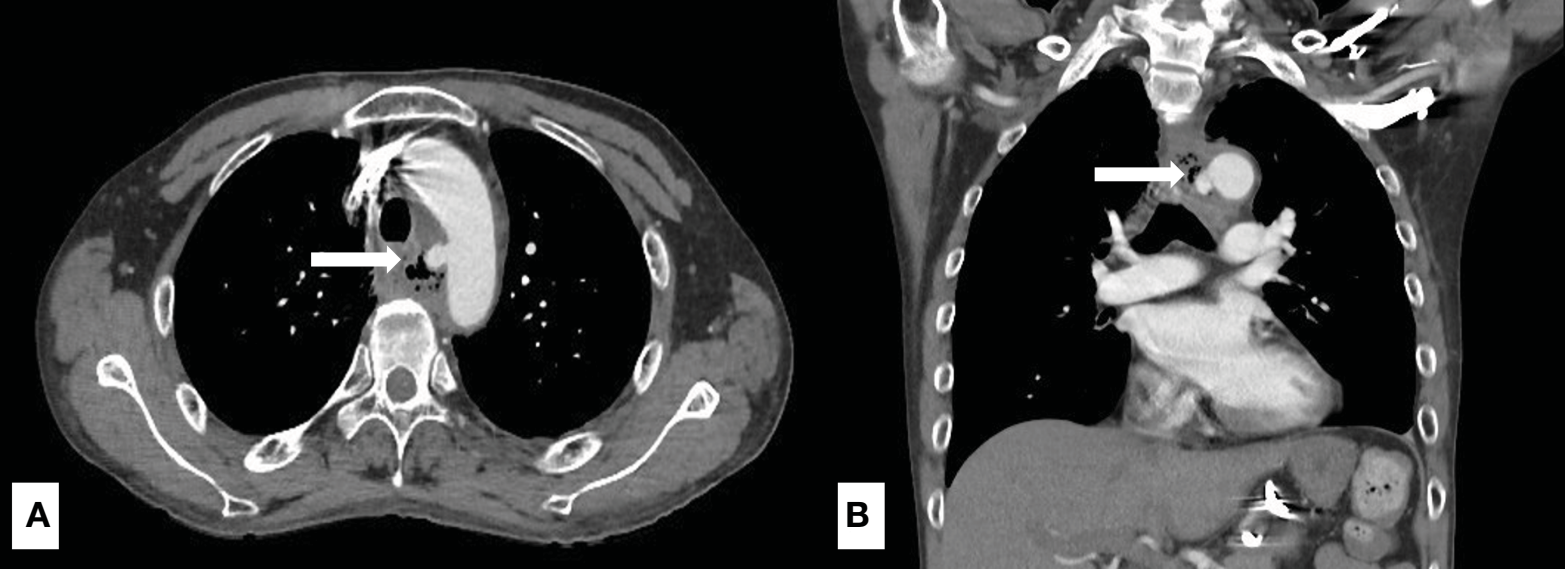
A 45-year-old male diagnosed with esophageal cancer post concurrent chemoradiotherapy. The computed tomography angiography of aorta revealed extravasation from the aortic arch abutting to esophagus (white arrow) with high suspicion of aorto-esophageal fistula in the axial view **(A)** and the coronal view **(B)**.

**Figure 2:**
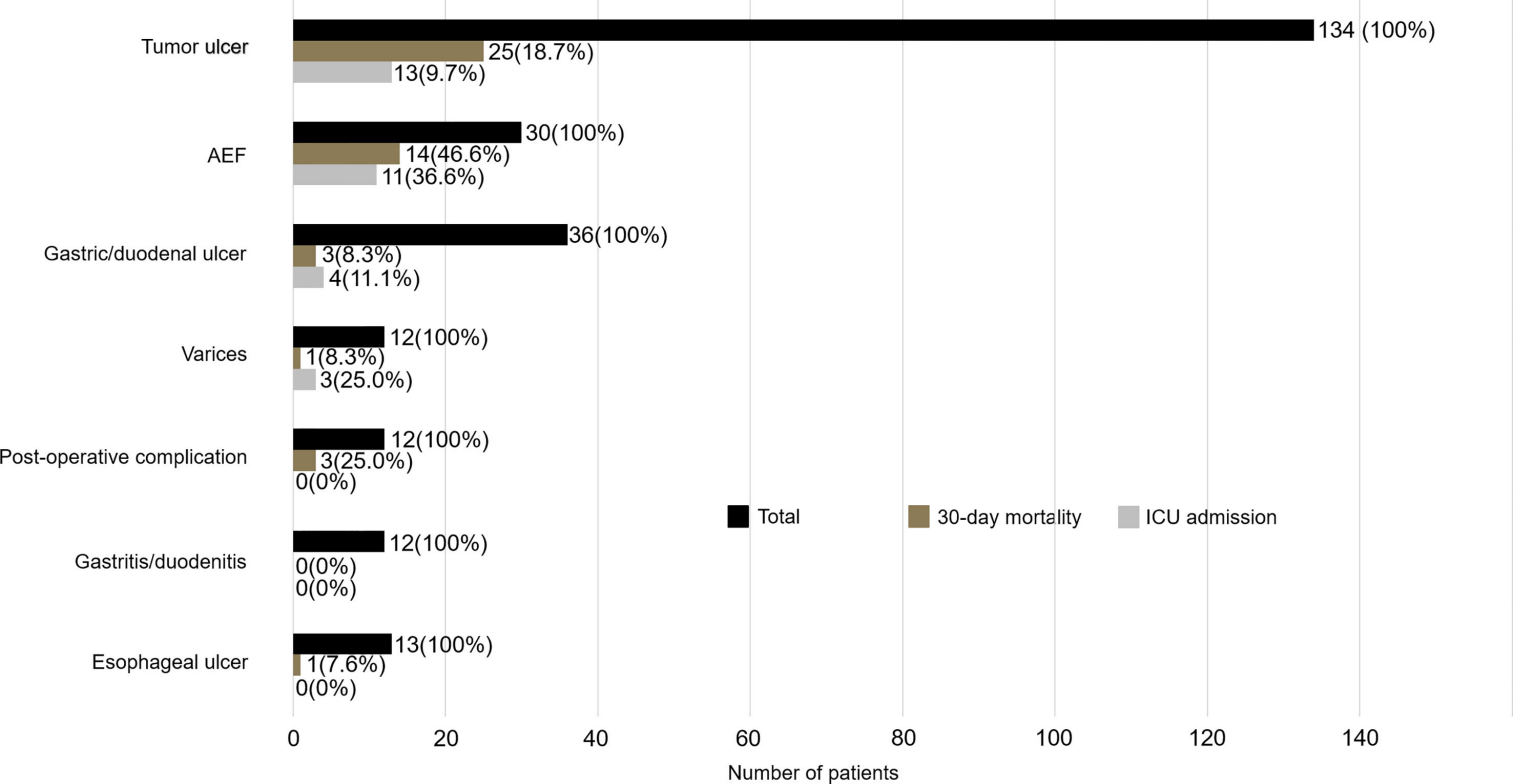
Summary outcomes with ICU admission and 30-day mortality stratified by confirmed etiologies.

**Figure 3:**
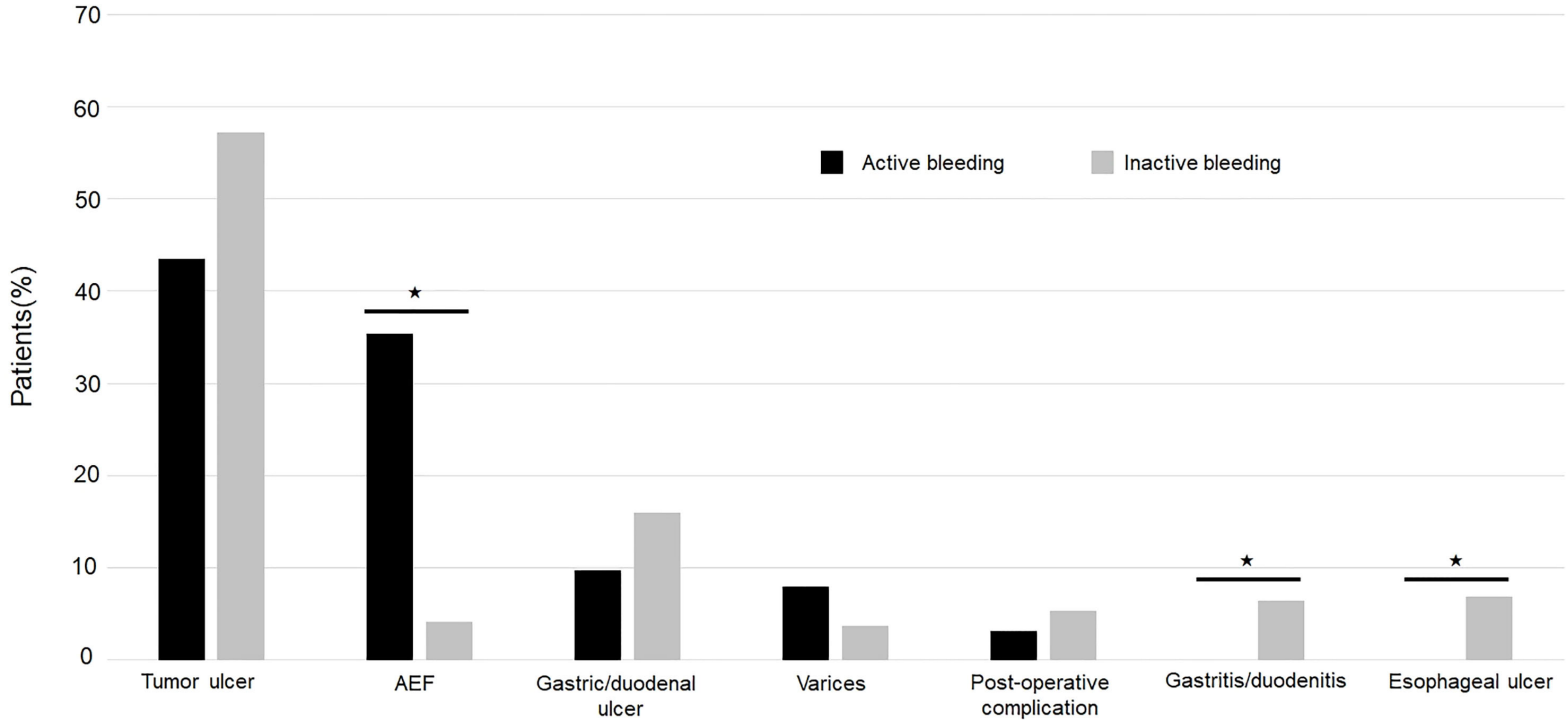
Distribution of UGIB etiologies categorized by active and inactive bleeding. *P<0.05.

Among all esophageal cancer patients with UGIB, 160 (64.2%) patients presented with hematemesis, and 89 (35.7%) patients presented with melena. 62 (24.9) patients were found to have active bleeding, and the proportion of active bleeding was significantly higher in the non-survivor group (51% vs. 18.8%, *p* < 0.001) (**Table 1**). The causes of UGIB were as follows: tumor-ulcer in 134 (53.8%) patients, gastric/duodenal ulcer in 36 (14.5), AEF in 30 (12.0%) (**Figure 1**), esophageal ulcer in 13 (5.2%), esophageal/gastric varices in 12 (4.8%), gastritis/duodenitis in 12 (4.8%), and postoperative complications in 12 (4.8%). Among the causes, AEF was significantly more frequent in the non-survivors than the survivors (29.7% vs. 7.9%, *p* < 0.001) (**Table 2**) and had the highest 30-day mortality rate (46.6%) and ICU admission rate (36.6%). Summary outcomes, with ICU admission and 30-day mortality of each etiology, were show in **Figure 2**. Additionally, we performed subgroup analyses on patients with active bleeding vs. non-active bleeding. The proportion of each etiology in the active bleeding group is as follows: tumor ulcer (27, 43.5%), AEF (22, 35.4%), gastric/duodenal ulcer (6, 9.7%), gastric/esophageal varices (5, 8.0%), and post-operative complication (2, 3.2%) (**Figure 3**). The proportion of AEF in the active bleeding group was significantly higher than in the non-active bleeding group (35.4% vs. 4.2%, *p* < 0.001).

Various treatments were performed for the management of UGIB as shown in **Table 2**. The non-survivor group showed significantly higher rates of blood transfusion (80.8% vs. 53.9%, *p* = 0.001), intubation (27.6% vs. 8.4%, *p* = 0.001), and inotropic agents support (10.6% vs. 0.9%, *p* = 0.003) than the survivor group.

### 3.3 Univariate and multivariate Cox regression analyses of 30-day mortality

Forty-seven (18.9%) patients expired after the follow-up period of 30 days. The causes of death were as follows: 15 (31.9%) patients due to aspiration pneumonia, 13 (27.7%) due to tumor-ulcer bleeding, 10 (21.3%) due to AEF, 4 (8.5%) due to end-stage tumor related multiple organ dysfunction syndrome, 2 (4.3%) due to severe sepsis, 1 (2.1%) due to gastric/duodenal ulcer bleeding, 1 (2.1%) due to post-operative anastomotic leakage, and 1 (2.1%) due to intracranial hemorrhage.

**Figure 4:**
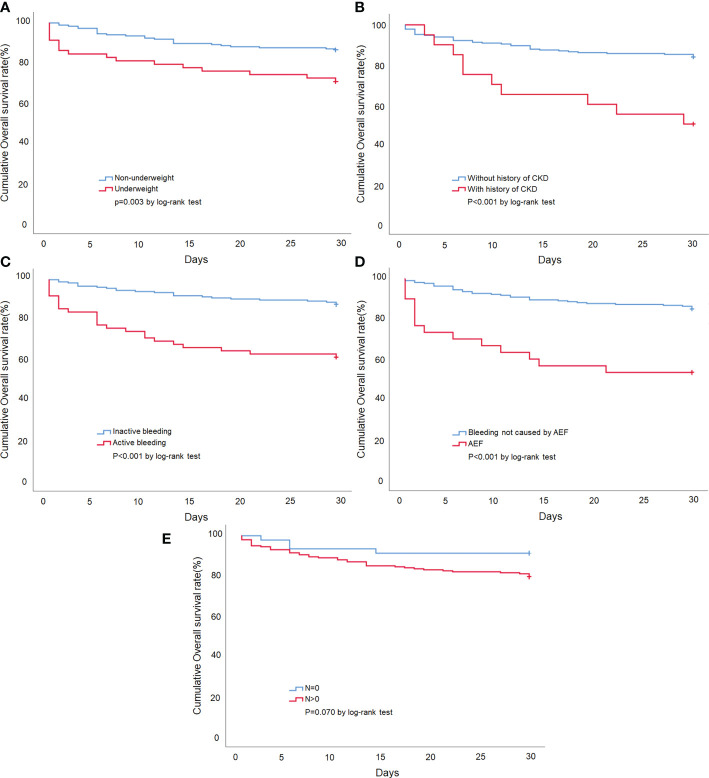
Kaplan-Meier survival curve of esophageal cancer patients presenting with UGIB. **(A)** Stratified by patients with or without underweight. **(B)** Stratified by the presence or absence of the underlying disease of CKD. **(C)** Stratified by patients with or without active bleeding. **(D)** Stratified by the bleeding etiology of AEF. **(E)** Stratified by tumor N stage >0 or tumor N stage =0.

Univariate and multivariate Cox regression analyses were used to investigate the prognostic factors for 30-day mortality (**Table 3**). The univariate predictors included underweight (HR = 2.45; CI = 1.32, 4.54; *p* = 0.004), ECOG-PS > 2 (HR = 2.84; CI = 1.37, 5.87; *p* = 0.005), underlying CKD (HR = 3.63; CI = 1.80, 7.32; *p* < 0.001), local recurrence (HR = 2.08; CI = 1.17, 3.69; *p* = 0.013), active bleeding (HR = 3.86; CI = 2.18, 6.85; *p* < 0.001), AEF (HR = 4.10; CI = 2.19, 7.68; *p* < 0.001), tumor N stage > 0 (HR = 2.74; CI = 1.03, 7.27; *p* = 0.042), blood transfusion (HR = 3.18; CI = 1.54, 6.58; *p* = 0.002), and WBC > 11 (10^3^/μl) (HR = 2.60; CI =1.47, 4.62; *p* = 0.001). The multivariate analyses indicated that underweight (HR = 2.02; CI = 1.02, 4.02; *p* = 0.044), underlying CKD (HR = 6.39; CI = 2.65, 15.4; *p* < 0.001), active bleeding (HR = 2.24; CI = 1.04, 4.82; *p* = 0.039), AEF (HR = 2.23; CI = 1.01, 4.92; *p* = 0.046), and tumor N stage > 0 (HR = 2.99; CI = 1.18, 7.57; *p* = 0.021) were statistically significant independent risk factors for 30-day mortality. We also performed a survival analysis for each independent risk factor, and the Kaplan-Meier survival curves can be seen in **Figure 4**.

**Table 3:** Univariate and multivariate analyses of predictors for 30-day mortality with Cox proportional hazards model.

	Univariate		Multivariate	
	HR(95%CI)	P value	HR(95%CI)	P value
Age	1.02 (0.99,1.05)	0.076		
BMI				
Non-underweight	Reference		Reference	
Underweight^〒^	2.45 (1.32,4.54)	0.004^*^	2.02 (1.02,4.02)	0.044^*^
ECOG-PS>2	2.84 (1.37,5.87)	0.005^*^	1.73 (0.70,4.26)	0.228
Heart rate>100 (beats/min)	1.76 (0.92,3.33)	0.083		
SBP<90 (mmHg)	1.55 (0.72,3.33)	0.256		
Underlying disease				
Hypertension	1.30 (0.69,2.28)	0.448		
Diabetes mellitus	0.75 (0.33,1.67)	0.475		
Coronary artery disease	0.96 (0.23,3.94)	0.949		
Congestive heart failure	0.48 (0.03,2.31)	0.540		
Chronic kidney disease	3.63 (1.80,7.32)	<0.001^*^	6.39 (2.65,15.4)	<0.001^*^
Prior cerebrovascular Accident	2.23 (0.88,5.65)	0.090		
Liver cirrhosis	0.66 (0.31,1.41)	0.280		
Local recurrence	2.08 (1.17,3.69)	0.013^*^	1.54 (0.79,2.98)	0.196
Active bleeding	3.86 (2.18,6.85)	<0.001^*^	2.24 (1.04,4.82)	0.039^*^
Arterial-esophageal fistula	4.10 (2.19,7.68)	<0.001^*^	2.23 (1.01,4.92)	0.046^*^
Anti-platelets agents use	1.02 (0.25,4.19)	0.981		
Anti-coagulant agents use	0.81 (0.11,5.90)	0.839		
Tumor stage				
T stage>1	6.04 (0.83,43.8)	0.075		
N stage>0	2.74 (1.03,7.27)	0.042^*^	2.99 (1.18,7.57)	0.021^*^
M stage>0	1.63 (0.90,2.97)	0.106		
Tumor treatment				
Surgical resection	0.64 (0.27,1.50)	0.300		
Chemoradiation	1.14 (0.57,2.29)	0.716		
Stent implantation	1.48 (0.77,2.86)	0.238		
Bleeding treatment				
Proton pump inhibitor	1.50 (0.36,6.19)	0.574		
Tranexamic acid	1.93 (0.98,3.78)	0.056		
Terlipressin	0.58 (0.29,1.45)	0.242		
Endoscopic treatment	0.80 (0.31,2.04)	0.643		
Surgical repair/Stent implantation	1.21 (0.50,2.87)	0.666		
Arterial embolization	0.59 (0.08,4.37)	0.614		
Blood transfusion	3.18 (1.54,6.58)	0.002^*^	1.25 (0.54,2.89)	0.592
Hospice care	1.47 (0.82,2.66)	0.194		
Initial Laboratory data				
WBC>11.0 (10^3^/μl)	2.60 (1.47,4.62)	0.001^*^	1.62 (0.80,3.26)	0.176
Hb<8.0 (g/dl)	1.22 (0.66,2.25)	0.522		
PLT<150 (10^3^/μl)	0.95 (0.50,1.80)	0.882		
PT>13 (sec)	1.55 (0.85,2.82)	0.151		
Cr>1.1	2.72 (1.51,4.91)	0.001^*^	1.17 (0.59,2.31)	0.638

HR: hazard ratio; 95% CI: 95% conﬁdence interval.

*P value < 0.05.

〒Underweight: BMI<18.5 kg/m2.

## 4 Discussion

To the best of our knowledge, this is the first and largest multicenter retrospective cohort study that evaluated the etiologies and risk factors for the adverse outcome in esophageal cancer patients with UGIB. The main findings of our results were that (1) the etiologies of esophageal cancer patients with UGIB included tumor ulcer (53.8%), gastric/duodenal ulcer (14.5%), and AEF (12.0%), (2) the ICU admission rate was 12.4%, and the 30-day mortality rate was 18.9%, (3) AEF (HR = 2.23, *p* = 0.046), underweight (HR = 2.02, *p* = 0.044), underlying disease of CKD (HR = 6.39, *p* < 0.001), active bleeding (HR = 2.24, *p* = 0.039), and tumor N stage > 0 (HR = 2.99, *p* = 0.021) were significant independent risk factors for 30-day mortality.

UGIB is a threatening situation that is underexamined in the current literature for esophageal cancer patients. Our findings revealed a mortality rate of 18.9% and an ICU admission rate of 12.4%, which highlight the complex and critical nature of the medical conditions these patients encounter. Previous studies illustrated that in the patients with UGIB, tumor bleeding was one of the etiologies with an incidence rate of 3.7%-5% (13, 14). The locations of tumor included the esophagus, stomach, and intestine. Esophageal cancer accounted for 0.7%-1.2% of these patients (13, 14). When compared to ulcer or variceal bleeding, esophageal cancer may seem to be a rare cause of UGIB. However, a previous study showed that in the gastrointestinal cancer patient population, 20% of UGIB was caused by tumor bleeding, which is notably higher than in the patients without a cancer history (15). In our study, tumor bleeding was the most common cause of UGIB, with a rate as high as 53.8% in the patients with esophageal cancer. This might be due to the fact that bleeding is a common complication in patients with advanced cancer (16, 17). Angiogenesis has been reported to be one of the features of advanced solid organ tumor—which includes esophageal cancer—and these blood vessels support the tumor for further growth and metastasis. Therefore, a high tendency of bleeding is expected due to the vessels’ structural fragiality (18). As esophageal cancer tends to be diagnosed at an advanced stage, tumor bleeding should be suspected in esophageal cancer patients with UGIB.

AEF is an uncommon and extremely fatal disease (19, 20), while only a few large studies have examined its etiology, risk factors, and prognosis owing to the rarity. Shinsuke et al. indicated that the most common cause of AEF is the post-operative status for aortic disease (40.6%), followed by primary aortic aneurysm (30.0%), bone ingestion (16.6%), and thoracic cancer (15.3%) (21). In the present study, 30 (12%) patients were diagnosed with AEF caused by esophageal cancer, and 14 patients died during the hospital stay. The high rate of AEF in our study may be due to factors like advanced cancer stage, chemoradiation therapy, and esophageal stents. Other possible causes, like infections or surgical complications, could also play a role in some cases (22). The mortality rate of AEF in our study was 46.6%, which was similar to the rate reported in the previous studies (47%-63%) (19, 20, 23). In our study, we found that AEF had the highest 30-day mortality rate and ICU admission rate when compared to the other groups. Moreover, AEF accounted for more than 10% of bleeding in this patient population, which is not rare when compared to the general population. Advanced esophageal cancer posed an increased risk of fistula formation, and in our cohort, 89.6% of the patients had locally advanced cancer (22, 23). Our study provided the first report of the incidence rate of AEF—12%—in the esophageal cancer patients with UGIB. Clinical manifestation of AEF has been classically described as Chiari’s triad, which is characterized by a sudden painful chest pain radiating to the back, followed by sentinel hemorrhage, and fatal exsanguination after an asymptomatic period (23–25). In our study, 30 patients were diagnosed with AEF, and 22 patients manifested with massive hematemesis. It is worth noting that Chiari’s triad is not constant, one study indicated that only 65% of the AEF patients have sentinel bleed, and only 59% of the patients recalled a history of chest pain (25). Therefore, AEF should be suspected in any patient with esophageal cancer bleeding who experiences rapid deterioration of hemodynamic status. In sum, AEF was an independent risk factor for 30-day mortality in our study, and prompt radiological diagnosis, such as contrast-enhanced chest CT, is critical in patients presenting with the aforementioned symptoms (23–25).

The association between AEF and mediastinal radiotherapy in advanced esophageal cancer patients is a significant concern, as radiotherapy can contribute to the development of AEF. Radiotherapy can cause local inflammation, fibrosis, and vascular injury, leading to the weakening of the esophageal and aortic walls. This, in turn, may result in the formation of a fistula, especially when the esophageal tumor erodes through the aortic wall (26–28). The combination of thoracic endovascular aortic repair (TEVAR) and esophageal self-expandable metal stents (SEMS) has emerged as a promising treatment strategy, as it addresses both the vascular and esophageal components of the fistula. TEVAR can help prevent further aortic wall erosion and stabilize the aortic wall, reducing the risk of fatal hemorrhage. SEMS, on the other hand, can provide immediate relief of dysphagia and facilitate the closure of the esophageal fistula by creating a barrier between the esophagus and aorta (29–31). Prophylactic TEVAR has been proposed as a potential preventive measure for AEF development in high-risk esophageal cancer patients undergoing mediastinal radiotherapy and SEMS placement. The rationale behind this approach is that early intervention with TEVAR may prevent aortic wall erosion and stabilize the aorta. However, the current evidence base is limited, and further research is needed to determine the optimal timing of TEVAR, patient selection criteria, and long-term outcomes (31–33).

The BMI is a widely used biomarker reflecting nutritional status due to its high accessibility. The association between BMI and the prognosis in patients with esophageal cancer has been undetermined. Some studies indicated that there was no correlation between BMI and the prognosis, while others stated that the outcome in the patients with low BMI is significantly poorer (34–36). Our study demonstrated that being underweight (BMI<18.5 kg/m^2^) was an independent risk factor for 30-day mortality. Nutritional status is an important to esophageal cancer patients. Poor intake due to dysphagia, odynophagia, and esophageal stenosis can lead to malnutrition and cachexia, which might weaken the immune system and reduce the resistance to infection. Additionally, previous research suggested that patients with nutritional deficiency might have poorer tolerance to the toxicity of chemoradiotherapy and surgical intervention (37–39).

Prior literature has indicated that patients with CKD showed an increased risk of cancer death (40–42). Samuel et al. found that the overall cancer death would increase by 18% for every 10-mL/min/1.73 m^2^ reduction in eGFR (40). Another study conducted by Weng et al. showed that the mortality rates in kidney, liver, and urinary tract cancers are inversely associated with renal function (41). We may speculate that the CKD patients usually have multiple comorbid conditions, and the deterioration of the underlying diseases during stressful events, such as UGIB, commonly occurs. Similarly, one study illustrated that the all-cause in-hospital mortality rate of UGIB in CKD patients is trifold higher than those without CKD (43). Moreover, patients with CKD often exhibit dysfunction of hemostasis, aggravating the bleeding condition in cancer patients (44, 45).

There were nearly a quarter (62, 24.9%) of the patients manifested with active bleeding in our study. Previous studies have shown that UGIB patients presenting with active bleeding have a tendency to develop unfavorable short-term outcome due to an elevated rate of recurrent bleeding and treatment failure (46, 47). Active bleeding might cause hemodynamic instability and render the clinical condition more challenging. In addition, over a third (22, 35.4%) of esophageal cancer patients with active bleeding were diagnosed with AEF, and a high mortality rate of 46.6% in our subgroup analysis was observed. As a result, AEF is uncommon in the general population but not a rare cause of bleeding in esophageal cancer patients with active UGIB.

We identified a tumor N stage > 0 as an independent risk factor for 30-day mortality. Several studies have demonstrated that metastatic lymph nodes were one of strongest predictors for poor survival rate in esophageal cancer (48–50). One study revealed that patients with 0, 1, and ≥ 2 lymph nodes metastasis had the 5 year-survival rates of 59.8%, 33.4% and 9.4% (49). The explanation for our result may be that lymph node metastasis itself has been shown to be associated with poorer prognosis in this population. Metastatic lymph nodes are commonly adjacent to large vessels. Similar to the formation of tracheal-esophageal fistula, AEF could develop after lymph node necrosis and vessel erosion, affecting the chance of survival in patients with esophageal cancer (22).

We recognized several limitations of the current study. First, the study was retrospective, and bias may exist because we could not control the accuracy and precision of the retrospective data. For example, patients with hematochezia, which can occur with massive UGIB, were excluded from the study because lower gastrointestinal bleeding was confirmed by endoscopy or imaging. The symptoms of GIB described by the patients was subjective. It was not plausible to limit recall bias, such as the quantity, frequency, or nature of bleeding. Personal lifestyle factors or previous medical history might be omitted by the patients. Second, the study was a single country study, and our patient population was mainly composed of esophageal SCC. Our results may not be extended to regions where the incidence of adenocarcinoma exceeds that of SCC. Third, while CT scans and endoscopies are commonly utilized diagnostic tools to determine the source of bleeding, the intermittent nature of bleeding can pose a challenge in accurately identifying the exact location of the bleeding site, leading to a missed diagnosis. Finally, despite this study being the largest to investigate UGIB in esophageal cancer patients, the small number of specific etiologies still limits the applicability of our findings. Further prospective multicenter studies with larger sample sizes are necessary to confirm our findings.

## 5 Conclusion

In esophageal cancer patients with UGIB, the most common etiology is tumor ulcer, followed by gastric/duodenal ulcer, and AEF. AEF accounted for 12% of UGIB in these patients, and was associated with the highest mortality rate. The index of suspicion for AEF is the vital signs of such patients, and prompt radiological diagnosis is required in patients with rapid deterioration in hemodynamic status or active bleeding. Underweight, underlying CKD, active bleeding, AEF, and metastatic lymph nodes were independent risk factors for the 30-day mortality in esophageal cancer patients with UGIB. The results of this study may enhance the physicians’ ability to perform risk stratification and select the optimal management method.

## Data availability statement

The raw data supporting the conclusions of this article will be made available by the authors, without undue reservation.

## Ethics statement

The studies involving human participants were reviewed and approved by Chang Gung Medical Foundation Institutional Review Board (IRB no. 202201321B0). Written informed consent for participation was not required for this study in accordance with the national legislation and the institutional requirements.

## Author contributions

Conceptualization, S-WL, C-PP, and C-CY. Data curation, T-HY and C-CY. Formal analysis, J-XL and C-CY. Investigation, C-HH and C-CY. Resources, J-XL and C-CY. Supervision, C-CY. Writing – original draft, S-WL and C-PP. All authors contributed to the article and approved the submitted version.
